# In vitro and in silico analysis of the anti-proliferative effects of *Spirulina platensis* on A549 lung cancer cells

**DOI:** 10.1038/s41598-025-24051-2

**Published:** 2025-11-07

**Authors:** Fatma I. Abo El-Ela, Marwa A. Ibrahim, Salma I. El-Samannoudy, Walid Hamdy Hassan, Doaa R. I. Abdel-Gawad

**Affiliations:** 1 Department of Pharmacology, Faculty of Veterinary Medicine, Beni-Suef University, Beni-Suef, 62511 Egypt; 2 Department of Biochemistry and Molecular Biology, Faculty of Veterinary Medicine, Cairo University, 12211 Giza, Egypt; 3 Department of Physiology Faculty of Veterinary Medicine, Cairo University, 12211 Giza, Egypt; 4 Bacteriology, Mycology and Immunology Department, Faculty of Veterinary Medicine, Beni-Suef University, Beni-Suef, 62511 Egypt; 5 Department of Toxicology and Forensic Medicine, Faculty of Veterinary Medicine, Beni-Suef University, Beni-Suef, 62511 Egypt

**Keywords:** Lung cancer, *S. platensis*, Cytotoxic, Epigenetics, Anti-oxidant, Molecular docking, ADMET, *In silico* study, Biochemistry, Cancer, Computational biology and bioinformatics

## Abstract

*Spirulina platensis* (*S. platensis*) is a natural microalgae extract that exerts significant cytotoxic effects against different serious cancerous diseases. Lung cancer is one of the most common and leading causes of death all over the world. The purpose is to investigate the anti-proliferative cytotoxic effects of *S. Platensis* against the adenocarcinomic human alveolar basal epithelial cells (A549 cell line). Also, molecular docking Analysis and pathway Map were investigated. The viability of the cells was determined *via* the MTT assay. Moreover the lipid peroxidation, total thiol, the protein concentration of Microtubule-associated tumor suppressor 1 (MTUS1), P16, Kirsten rat sarcoma viral oncogene homolog (K-ras), the epidermal growth factor receptor (EGFr), and the molecular parameters (short stature homeobox 2 (SHOX2), Breast cancer metastasis suppressor 1 (BRMS1), B-cell lymphoma 2 (BCL-2), and Bcl-2-associated X protein (BAX) were assessed. In order to define interaction sites and classes, the current study investigates in detail the interactions between the ligand *S. platensis* and the Interleukin enhancer-binding factor 3 Receptor (IL-F3) of lung cancer. Covalent bonds, H-bonds, and hydrophobic interactions were observed to form with critical residues on the active site. Covalent bonds are identified in seventeen of the complexes. A correlation was observed between binding affinity and molecular size, branching, polar surface area of up to 199 Å2, hydrophilicity, and topological diameter for various bonds. In an effort to enhance pharmaceutical quality control, we utilized *in silico *methodologies to forecast the ADMET (absorption, distribution, metabolism, excretion, and toxicity) of *S. platensis*. Results revealed a great anti-proliferative cytotoxic effect with a concentration gradient through decreasing the peroxidation content, and the epigenetic markers, with significant up-regulations of the BRMS1 and BAX. In addition to the kinetic and MAP pathways, docking analysis confirmed that *S. platensis* binds with the highest affinity to the predicted active sites of the tumor receptor IL-F3, thereby validating its antitumor activity. In conclusion, great suppression in the virulence of lung cancer was reported following treatment with *S. platensis*, illustrating its suspected mechanism of action, safety profile, kinetic properties, molecular docking results, toxicity, and internal pathways.

**Supplementary Information:**

The online version contains supplementary material available at 10.1038/s41598-025-24051-2.

## Introduction

 Lung cancer is considered the leading cause of cancer-related deaths^1^ and one of the most common cancers worldwide^2^, affecting both males and females^3^. Small cell lung cancer (SCLC) and non-small cell lung cancer (NSCLC) are the main types of lung cancer^4^. The NSCLC accounting for more than 85% of cases are related to NSCLCs^1^.

Exposure to radioactive substances and detrimental radiation, smoking, chewing, and tobacco are the predisposing factors for lung cancer^5^. The interaction between the environmental, genetic, and epigenetic factors is the cause for lung carcinoma initiation and progression^6^. The genetic alterations may be occurring at the chromosomal level, in certain genes, either by addition or deletion, structural changes or at the expression level^7^. The most common epigenetic changes in lung cancer are the promotion of DNA methylation that results in gene silencing. These epigenetic changes may be happen at certain nuclear positions and chromosome domains following environmental exposure to the previous predisposing factors^6^.

Detection of lung cancer may happen accidentally during any routine examination or any other related respiratory disorders^8^. The therapeutic interventions differ from case to another according to the stage of the disease it may be targeted therapy, immunotherapy, chemotherapy, radiation or surgical intervention^9^.

Recently, many pharmaceutically synthesized products that are used in cancer therapy have been replaced by natural ones to avoid its toxic or negative side effects^10^, as pain, alopecia, weight loss, cytopenia, hepatotoxicity, nephrotoxicity, cardiac weakness, nausea, diarrhea, impairment, and anaphylaxis that resulted from chemotherapy^11^. The chemotherapeutic agents may affect them negatively on the normal cells^12^.

Paclitaxel (Taxol^®^) ^13^and Curcumin (diferuloylmethane) are examples of drugs used in cancer therapy of plant origin^14^. The low bioavailability of curcumin resulted in the use of high concentration or in combination with other formulations^15^. Camptothecin, paclitaxel (Taxols), and topotecan (Hycamtins) are antitumor synthetic analogs derived from plants, while other forms could be derived from earth bacteria, as anthracyclines doxorubicin (Doxils; Adriamycins), glycopeptide bleomycin (Blenoxanes), and the nonribosomal peptide dactinomycin (Cosmegens)^16^.

One of the naturally derived therapies is *spirulina* extracts that have proved to suppress variant types of cancer^17^, through inhibiting the cellular proliferation and migration with induction of apoptosis and cell cycle arrest^18^. The cytotoxic effects of *spirulina* were reported against lung cellular^19^, hepatocellular and colon carcinoma^20^, human acute and chronic myelogenous leukemia^21^, human chronic myelogenous^22^, breast cancer^23^, and bone marrow cancer^24^.

Arthrospira platensis or *spirulina*, as generically named, belongs to the family Microcoleaceae^25^, phylum of cyanobacteria^26^. These blue-green microalgae are rich in the natural bioactive substances^27^, mainly the granular and the antioxidant substances as chlorophyll, phycocyanin, beta-carotene, xanthophyll, myxoxanthophyll, and zeaxanthin, also minerals, vitamins, amino acids, and proteins^28^. These bioactive substances enable it to be incorporated widely in the food^29^, and pharmaceutical industry^30^. It has several activities such as anti-inflammatory^31^, anti-microbial, anti-aging, anti-oxidants, anti-cancer^32^, and activating the metabolism of lipids and glucose, and the immune system^33^.

Interleukin enhancer binding factor (ILF), which is alternatively referred to as nuclear factor 45 (NF45), is produced by a locus situated on chromosome 1 of the human genome. Interleukin 45 is another name for interleukin enhancer binding factor (ILF). ILF contributes to the progression and development of breast cancer and other forms of cancer^34^. Regarding malignancies, ILF has been the subject of extensive research in recent years. ILF, for instance, is upregulated in numerous malignancies, such as glioma, NSCLC, and esophageal cancer, and contributes to their progression ^35,36^. Consequently, ILF upregulation might be required for the progression of cancer cells. Furthermore, ILF expression is correlated with the extent of pancreatic ductal carcinoma (PDAC) tumors, according to recent research ^37,38^. Moreover, ILF may serve as a significant prognostic indicator for the survival of PDAC^38^. ^39^ reported that ILF2 expression is significantly elevated in liver cancer tissues, according to immunohistochemistry and western blotting data. The precise function of ILF and the mechanism by which it contributes to tumorigenesis, nevertheless, necessitates additional research. Therefore, for complete clearance, molecular docking studies with in silico toxicity, safety, and pathway map investigation are crucial for determining the interaction of *spirulina* with this receptor type.

In the current study, the binding efficacy of *S. platensis*, which has been previously synthesized and experimentally bound to the ILF2 receptor, is evaluated using the binding free energy (ΔG) and binding affinity. The binding site and interaction were meticulously depicted, and an exhaustive search was conducted for the factors that enhance these interactions and improve the binding efficacy. Consequently, comprehending the factors that reinforce these interactions, in addition to their binding sites and interactions, aids in the identification of potentially effective pharmaceuticals from the vast pool of natural products and repurposed drug candidates. An in silico toxicity investigation, computational chemistry, or molecular docking can be of great assistance in determining the ligand-receptor binding reactivity. Additionally, the identified binding sites and mechanisms of action were compared to carboplatin, a standard anticancer agent used to treat lung cancer.

From this work, we aimed to estimate the anti-proliferative cytotoxic effect of *S. platensis* against A54 lung cancer cell line which will provide a potential efficient therapy for this lethal cancerous type. In addition to a clear mechanism of action and distinct pathway for the anticancer activity of the *S. platensis* with safety, molecular docking and in silico toxicological investigations.

##  Materials and methods

###  Method of *Spirulina platensis* microalgae preparations

Once the green microalgae *Spirulina Platensis* were acquired, they were gathered and maintained in an alkaline medium pH. *Spirulina* growth typically transpired during the summer. Following this, the pond water was drained and the *spirulina* were gathered in a silk cloth equipped with micro-sized apertures. The algae were subsequently filtered through these apertures; this procedure was iterated multiple times, and the resulting material was passed through smaller holes.

The Amoun Veterinary Company (AVC) in Cairo, Egypt, supplied the green pulverized form of *S. platensis* for the synthesis of pharmaceutical medications as a pure green powder. Samples are collected from prepared green *spirulina* for the subsequent analyses: protein content (56.5%), fatty acid composition (Caprylic acid (1.6%), Capric acid (34.62%), Myristic acid (0.41), tetraacdecenoic acid (0.58%), pentaadeconeic acid (0.25), palmetic acid (24.64%), linoleic acid (24%), and vitamin, mineral, and amino acid analysis (along with 98% carotenoids.)

###  Cell culture

A549 Cell lines (VACSERA, Cairo, Egypt) were used for determination the anti-proliferative cytotoxic effect of *S. platensis*, the cells were cultured in DMEM medium enhanced with FBS (10%), penicillin (100 units/mL), and of streptomycin (100 mg/mL) and kept in a humidified atmosphere (5% CO_2_ at 37 °C).

### Ethical approval

The in vitro procedures of the current study were done according to the standards set forth guidelines for the care and use of experimental animals by the Institutional Animal Care and Use Committee of Vet. CU. IACUC, Cairo University (Vet CU 03162023594).

### Samples

A stock solution of the powdered sample of *S. platensis* was pre-solubilized in dimethylsulphoxide (DMSO) at 37 °C. Serial two-fold dilutions were made, working concentrations of 100, 50, 25, 12.5, 6.25, and 3.125 µg/mL.

###  MTT cytotoxicity assay

A confluent monolayer of A549 cells was cultivated in 96 microtiter plates for about 24 h. The cells were incubated with different concentrations of the *S. platensis* in triplicate at 37 °C in a CO_2_ environment for 24 h. then, carefully added 20µL 5 mg/mL MTT to each well and incubated at 37 °C for 4 h. Then, remove the media gently with the addition of 150µL MTT solvent. Cover with tinfoil and agitate cells on orbital shaker for 15 min. Finally, the OD was measured at 570 nm in a microplate reader (BMGLABTECH^®^FLUOstar Omega, Germany).

The Inhibitory concentration (IC_50_), was estimated from the graphic plots of the dose-response curve for each conc. Using GraphPad Prism software (San Diego, CA. USA).The cell line was treated with the *S. platensis* at a concentration of calculated the IC%50 for 24 h., then centrifuged to collect the cells and obtain the supernatant for further analysis.

### Determination the content of lipid peroxidation (LPO) and total thiol

The method of ^40^ was adopted for estimation of total thiols content; in contrast, the MDA content was determined according to the method described by ^41^.

###  Determination the concentration of MTUS1 and P16 protein* via* ELISA

The concentration of both MTUS1 and P16 proteins was determined in the supernatant through the quantitative sandwich ELISA technique according to the instructions of the abbexa ELISA kit (Cat. No.: abx391613) for MTUS1 and My BioSource ELISA kit (Cat. No.: MBS043874) for P16.

### Determination the total protein content of K-ras and EGFR *via* Western blot technique

Kit of the ReadyPrepTM protein extraction (total protein) supplied by Bio-Rad Inc (Catalog #163–2086) was used in relation to the manufacturer’s instructions. It was added to each sample of the lysed cells of the control (A549) and *S. platensis* treated cancerous cell lines. The quantitative protein analysis in each sample was done *via* Bradford Protein Assay Kit (SK3041) following the instructions of the manufacturer (Markham Ontario L3R 8T4 Canada). Polyacrylamide gels were implemented using TGX Stain-Free™ FastCast™ Acrylamide Kit (SDS-PAGE), which was delivered by Bio-Rad Laboratories Inc Cat # 161–0181. The SDS-PAGE TGX Stain-Free FastCast was made following the instructions of the manufacturer. The gel was structured in a transfer sandwich as follows from below to above (filter paper, PVDF membrane, gel and filter paper). The sandwich was set in the transfer tank with 1x transfer buffer, which is composed of 190 mM glycine, 25 mM Tris and and 20% methanol. For allowing transfer of the protein bands from gel to the membrane, the blot was run for 7 min at 25 V by BioRad Trans-Blot Turbo. The membrane was blocked at room temperature for 1 h in Tris-buffered saline with 3% bovine serum albumin (BSA) and Tween 20 (TBST) buffer. The blocking buffer was composed of 150 mM NaCl, 20 mM Tris pH 7.5, 3% bovine serum albumin (BSA) and 0.1% Tween 20. Depending on the manufacturer’s instructions, the purchased Primary antibodies of K-ras and EGFR were diluted in TBST. Each primary antibody solution was incubated overnight against the blotted target protein at 4 °C. Followed by rinsing the blot 3–5 times for 5 min with TBST. Incubation was done in the HRP-conjugated secondary antibody (Goat anti-rabbit IgG- HRP-1 mg Goat mab -Novus Biologicals) solution against the blotted target protein for 1 h at room temperature. The blot was rinsed 3–5 times for 5 min with TBST. The chemiluminescent substrate (Clarity TM Western ECL substrate Bio-Rad cat#170–5060) was applied to the blot according to the manufacturer’s recommendation. Briefly, equal volumes were added from solution A (Clarity western luminal/enhancer solution) and solution B (peroxidase solution). The chemiluminescent signals were captured using a CCD camera-based imager. Image analysis software was used to read the band intensity of the target proteins against the control sample beta actin (housekeeping protein) by protein normalization on the ChemiDoc MP imager^42^.

### Quantitative assay of the m-RNA levels SHOX2, BRSM1, BAX, and BCL-2 genes

The total RNA was extracted using the RNeasy Mini Kit (Qiagen Cat. /ID 74104). SuperScript Reverse Transcriptase (thermoscientific) was then used to generate the first-strand cDNA in accordance with the manufacturer’s directions^43^. On an ABI Prism StepOnePlus Real-Time PCR System (Applied Biosystems), quantitative PCR was carried out using PowerTrack SYBR Green Master Mix in accordance with the manufacturer’s specifications. Table 1 contains the primer sequences designed for the target genes. Primer 3 software was used to design the primer sets^44^. Target mRNA expression was normalized to ACTB^45^, and the expression data were computed using the normalized fold change method^46^.

**Table 1:** Primers of the studied genes:

Gene	Forward primer	Reverse primer	Product	Accession no
BCL2	CCTCGCTGCACAAATACTCC	TGGAGAGAATGTTGGCGTCT	184	NM_000633.3
SHOX2	AAGCCAACGAAAGCTGAGGTC	AACCTGAAAGGACAAGGGCG	459	*NM_003030.4*
BAX	AAGAAGCTGAGCGAGTGTCT	GTTCTGATCAGTTCCGGCAC	458	NM_138761.4
BRMS1	GTACGAATGTGAGCTGCAGG	GTACGAATGTGAGCTGCAGG	539	NM_015399.4
ACTB	CATCCGCAAAGACCTGTACG	CCTGCTTGCTGATCCACATC	218	NM_001101

###  Molecular docking

#### Different websites used for ligand and protein receptor preparation

The interaction between *S. platensis* with the predicted examined protein on the lung cancer surface in the study, along with their associated 3D and 2D binding affinity, and illustrated bonds and interactions. The X-ray crystal structure of *S. platensis* from was obtained in 3D SDF form from the PubChem site (https://pubchem.ncbi.nlm.nih.gov/compound/3086069) and a homology model of Interleukin enhancer-binding factor 3 Receptor (ILF3 receptor) was obtained from the Uni-prot (https://www.uniprot.org/uniprotkb/Q12906/entry) and the protein data bank site (PDB ID Q12906· ILF3_HUMAN) besides confirmed its code and ID from the human gene bank on https://www.genecards.org/cgi-bin/carddisp.pl?gene=ILF3. These existing structures were used as targets for docking after determining the active site or pockets of interactions from the CB.DOCK site and confirmed also from the Deep site. The proteins’ 3D model structures were obtained from the UniProt KB database, improved using ModRefiner (https://zhanggroup.org/ModRefiner/) to increase the protein quality, and their active sites were predicted using Deep site server (https://www.deepsite.ai/) and confirmed with more accuracy by CB.DOCk (https://cadd.labshare.cn/cb-dock2/php/blinddock.php#job_list_load).

#### Preparation and computations of ligand

The ligands, specifically Spiraline (*Spirulina platensis*), were acquired in 3D-sdf format from the PubChem database. The energy-minimization process was performed on their 3D structures using Avogadro 1.2.0 software 25 with a force field of MMFF94. Following protein preparations, the ligand, which is currently stored in PDB format, will subsequently be analyzed on Auto Duck Vienna. This analysis will be conducted in pdbqt format to ensure that the ligand is prepared for visualization and interactions on the Biovia 2021 program.

#### Preparation and computations of protein receptor

In order to prepare proteins, a three-dimensional (3D) model of the ILF3 receptor was acquired.Uniprot-downloaded PDB structure with knowledge of all chain types, XRD, and alpha fold structure of the entire protein. Prior to docking, the ILF3 protein underwent preparation, which involved the removal of water molecules and the addition of hydrogen atoms to the unoccupied valences of the heavy atoms. After examining the protein’s structure to identify any missing atoms that required addition, any incomplete portions were repaired, and the KOLLMAN charges were added. The ILF3 protein was classified as a receptor, and the appropriate binding site (comprising active sites of interaction with the *S. platensis* ligand) (Pockets) was determined using the deep site and CB-DOCK server in conjunction with the Define and Edit Binding Site protocol. Docking simulations were executed utilizing the AutoDock Vina software, employing a 30 × 30 × 30 grid frame. Following the estimation and determination of active sites using a grid box measuring 30 × 30 × 30 along the x, y, and Z axes, the grid box was centered at the precise center of the active site. Subsequently, the active sites were identified using the exact amino acids obtained from the CB-Dock (P: 174; R:177; M:180; E:181; Y:784; L:207 & R:211; all in the A-chain). Once the determined active sites were confirmed, they were deleted. The dimensions of the grid box were documented for subsequent use in the configuration file, where the binding affinity between the ligand and receptor was calculated. In order to prepare the protein for docking with the ligand using the visualization DISCPVERY STUDIO (BIOVIA 2021) program, it was ultimately stored in pdbqt format as well.

#### Protein–ligand interactions

Once the binding affinity between the ligand and receptor had been determined, the server software was utilized to visualize and scrutinize the protein–ligand interactions. Subsequently, *S. platensis* was introduced in pdbqt format and designated as the ligand. The ILF3 protein was introduced in pdbqt format and designated as the receptor. By including all atom distances, angles, and amino acids in the desired format, interactions between the ligand and receptor were ascertained. H-bonding, hydrophobic interaction, salt bridge between covalent bonds, aromatic ring center, charge center, and π-stacking (parallel and perpendicular) were the interactions that were investigated. In order to evaluate the protein–ligand interaction, the binding affinity and free energy (ΔG) were computed. All interactions were captured in 3D and 2D at various dimensions in order to investigate interactions.

#### Kinetics process analysis and safety evaluations (Prediction of ADMET by computational analysis)

ADMET analysis (absorption, distribution, metabolism, excretion, and organotoxicity) was performed using ADMETlab 2.0 web server 26 (https://admetmesh.scbdd.com/), and Pathway MAP analysis was performed using the PLAY MOLECULES database on the server Pathway MAP (https://playmolecule.org/). In conclusion, a 2D and 3D analysis was conducted utilizing the BIOVIA web application in conjunction with the SMILES codes.

An initial examination was conducted on the targets of *S. platensis* in order to assess its potential anticancer properties *via* the Swiss Target Prediction server, which operates online. Compound PK properties, including absorption, distribution, metabolism, excretion, and toxicity (ADMET) profiling, were ascertained utilizing the ADMET descriptors algorithm protocol1 and the Discover Studio 4.0 (DS4.0) software package (Accelrys Software, Inc., San Diego, CA, United States) and the pkCSM (https://biosig.lab.uq.edu.au/pkcsm/). The lipophilicity levels expressed as atom-based LogP (AlogP98) and the 2D polar surface area (PSA_2D), which is a primary determinant of fractional absorption, are two significant chemical descriptors that exhibit strong correlations with PK properties. As indicated by the colon cancer cell line Caco-2, drug absorption is dependent on a variety of factors, including intestinal absorption, cutaneous permeability levels, P-glycoprotein substrate or inhibitor, and membrane permeability. Volume of distribution (VDss), permeability of the central nervous system (CNS), and the blood-brain barrier (logBB) are all determinants of drug distribution. Predictions of metabolism are generated using CYP models, which represent cytochrome liver enzymes involved in substrate or inhibitory processes (CYP2D6, CYP3A4, CYP1A2, CYP2C19, CYP2C9, CYP2D6, and CYP3A4). On the basis of the total clearance model and renal OCT2 substrate, excretion is anticipated. On the basis of AMES toxicity, hERG inhibition, hepatotoxicity, and cutaneous sensitization, the toxicity of drugs is predicted. PKCSM Prediction: http://biosig.unimelb.edu.au/pkcsm.

#### Toxicological investigations and safety estimations

The compliance of these calculated parameters with their respective standard ranges was verified. Pro-tox (Prediction of Toxicity of Chemicals) (https://tox.charite.de/protox3/) and Toxtree (AMBIT; https://apps.ideaconsult.net/data/ui/toxtree), Version 2.6.13 (Ideaconsult, Ltd., Sofia, Bulgaria), were utilized to assess the prognosis of genotoxicity and additional adverse effects. Both open source software and readily accessible in silico programs are utilized to detect chemical structural alarms (SA).

### Statistical analysis

The expression for the data is (mean values ± standard error (SE)). Continuous variables were examined utilizing the T test for independent samples. A P value less than 0.001 was deemed to be statistically significant. All analyses of the data were performed using SPSS version 9.0 (*n* = 3). A stepwise multiple regression analysis was conducted utilizing version 25 of the SPSS software. The correlation coefficient (R), standard error of the estimate (SE), number of data points (N), least significant difference (p), and 95% confidence intervals (enclosed in parentheses) for each regression coefficient were utilized to assess the validity of the models.

## Results

### Anti-proliferative cytotoxic effect of *S. platensis* on

#### Morphology

**Fig. 1:**
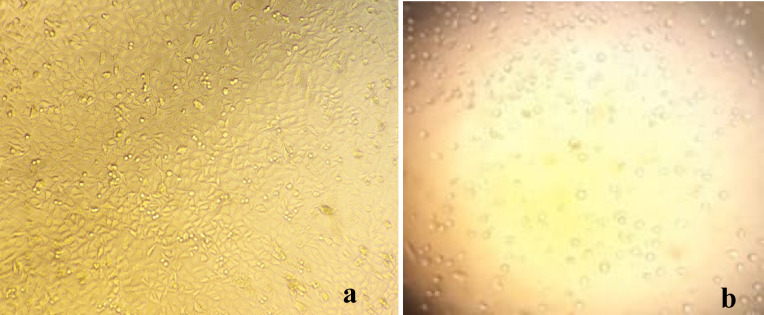
The morphology of A549 (**a**) control A549 cell line (**b**) cytotoxic effect of *S. platensis*.

The cytotoxic effect of *S. platensis* was reflected morphologically on the shape of the cells (Fig. 1b), in which the cells appear shrunken and pebble-shaped that differs from the shape of the untreated cells (Spindle shape adherent cells resembling the squamous epithelial cells) (Fig. 1a).

#### Viability %

**Fig. 2:**
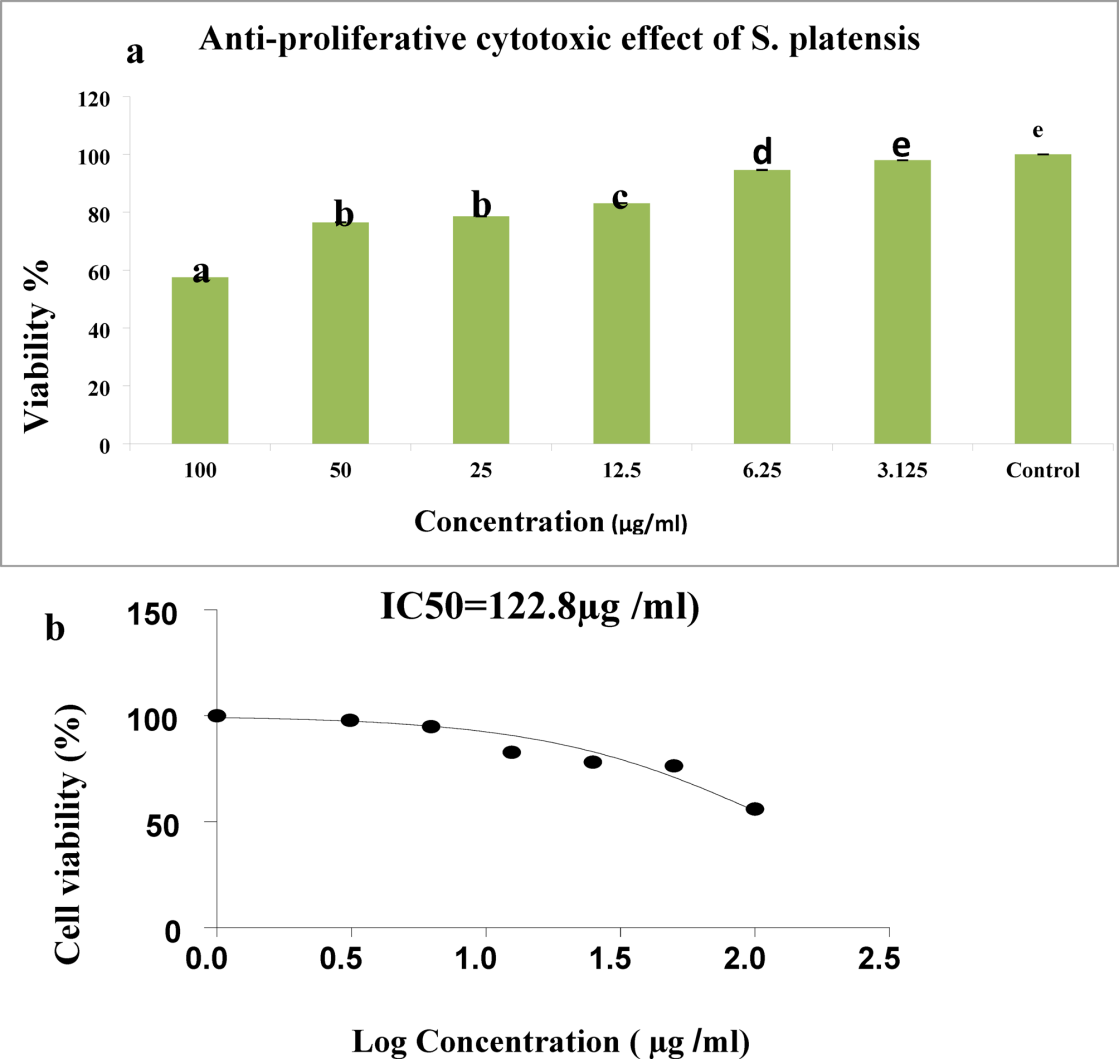
(**a**)Anti-proliferative cytotoxic effect of *S.platensis* on A549 cell line at different tested concentrations, Fig. ( **b**): IC_50_ of *S.platensis* on A549 cell line.

Figure 2a illustrates that *S. platensis* exhibited a potent anti-tumor cytotoxic effect against A549 cell lines, where the survival fraction showed a significant decrease as the concentration of *S. platensis* increased. The IC50 value was reached for the examined substance (122.8 µg/mL) (Fig. 2b).

### The content of lipid peroxidation (LPO) and total thiol

**Fig. 3:**
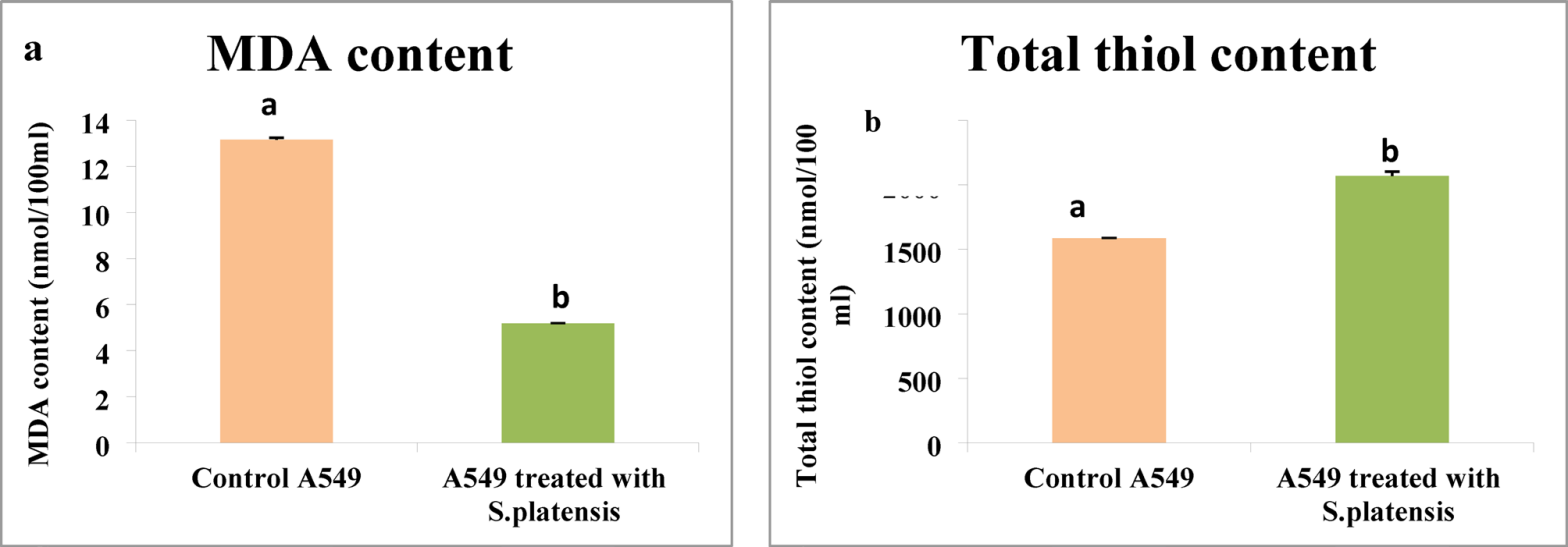
(**a**) MDA content in control A549 and the tretaed cell line with *S. platensis*, (**b**): Total thiol content in control A549 and the treated cell line with *S. platensis.*

In comparison, the treated and untreated lung cancer cell line with *S. platensis* the MDA content was significantly decreased in the treated one as shown in Fig.3a, moreover, the total thiol was significantly increased following treatment Fig. 3b.

### The concentration of MTUS1 and P16

**Fig. 4:**
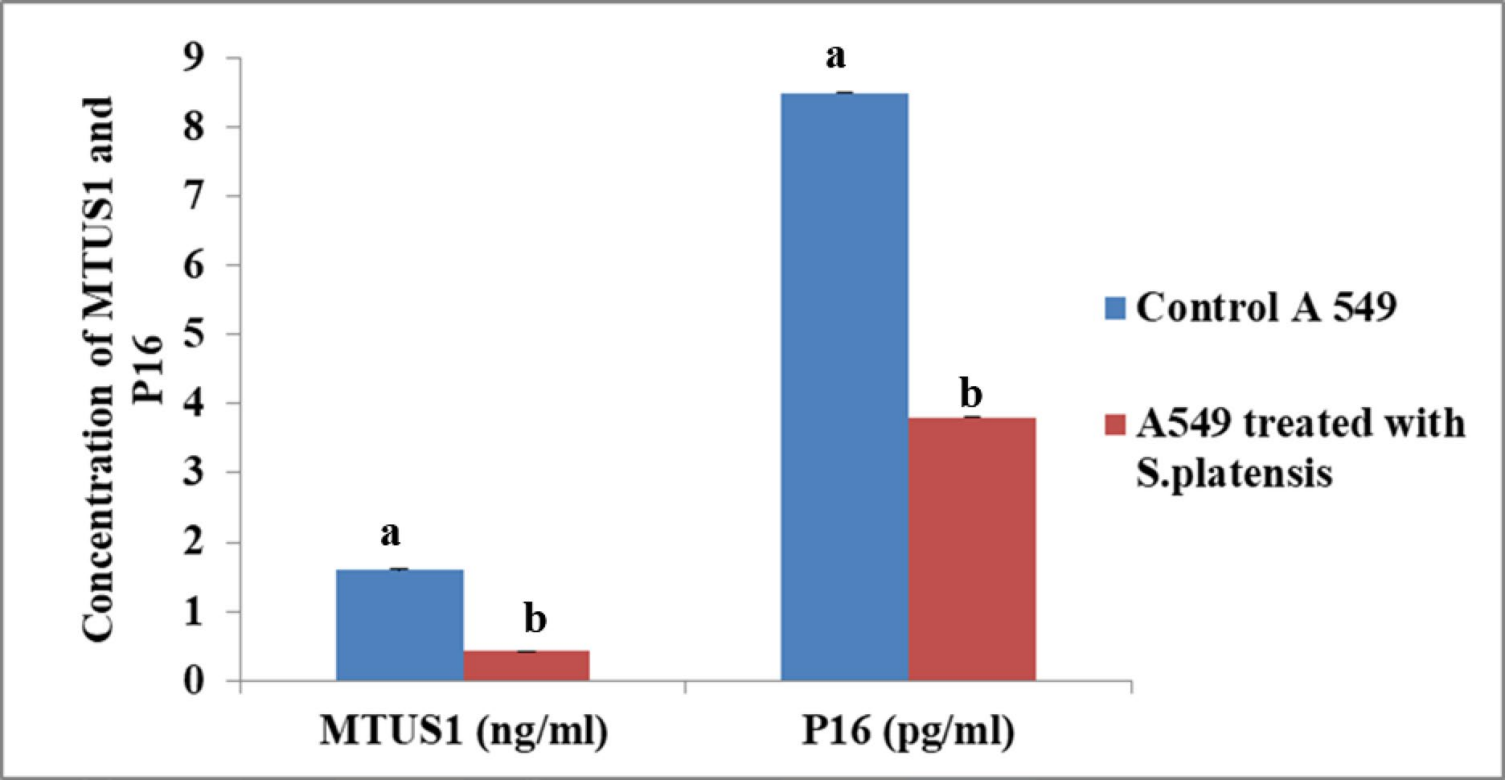
MTUS1 and P16 concentration in the control A549 and the tretaed cell line with *S. platensis*.

A549 cells treated with *S. platensis* was exhibited a significantly lower concentration of MTUS1 and P16 significantly in comparison to the non-treated A549 cell line as shown in Fig. (4).

### The protein concentration of K-ras and EGFr

**Fig. 5:**
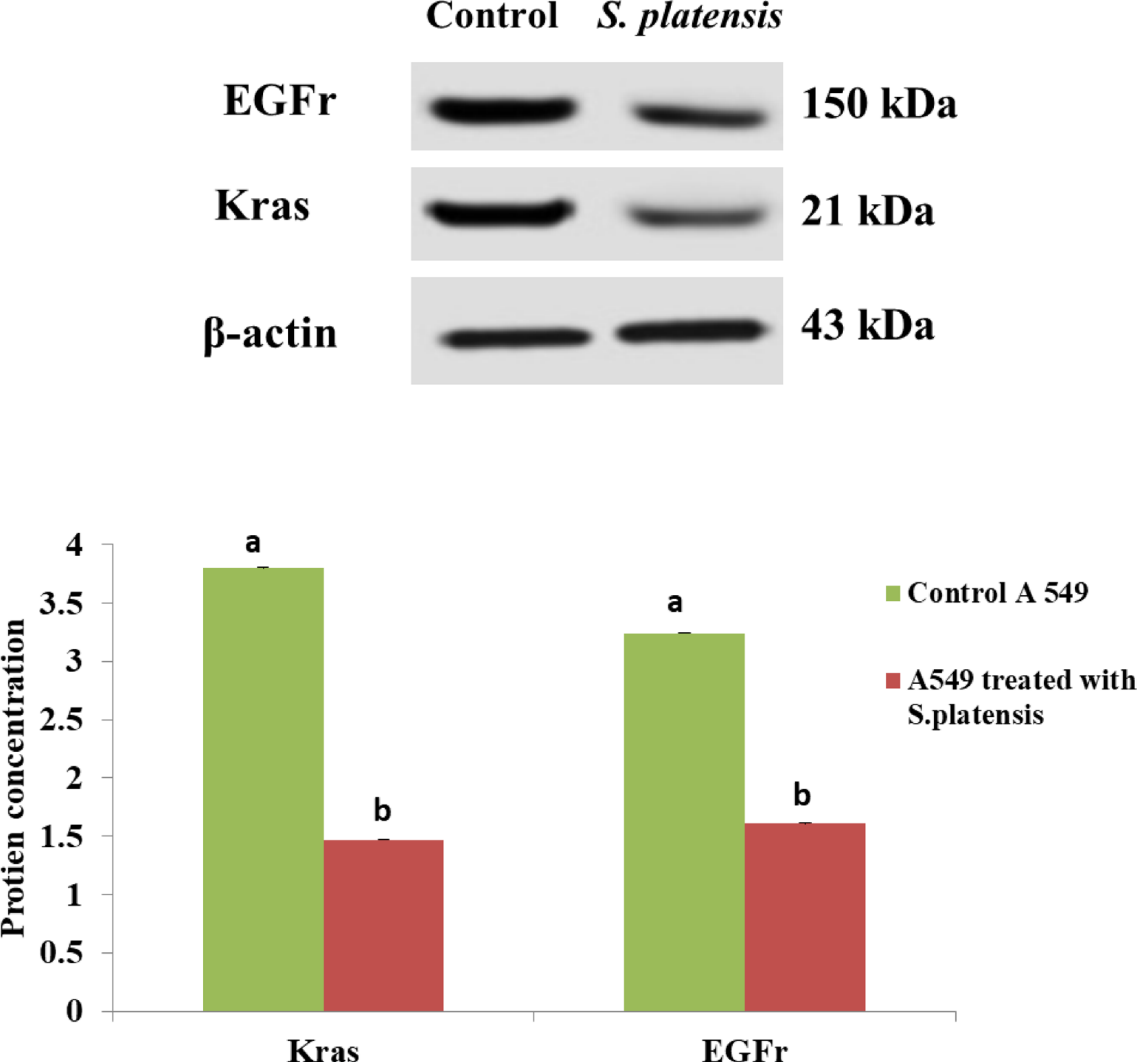
K-ras and EGFr concentration in the control A549 and the treated cell line with *S. platensis*.

Figure 5 was illustrated that the K-ras and EGFr concentration was significantly reduced in A549 cell line treated with *S. platensis*. The uncropped blot/gel images were provided as supplementary files (Fig S1,S2).

### The m-RNA levels SHOX2, BRSM1, BAX, and BCL-2 genes

**Fig. 6:**
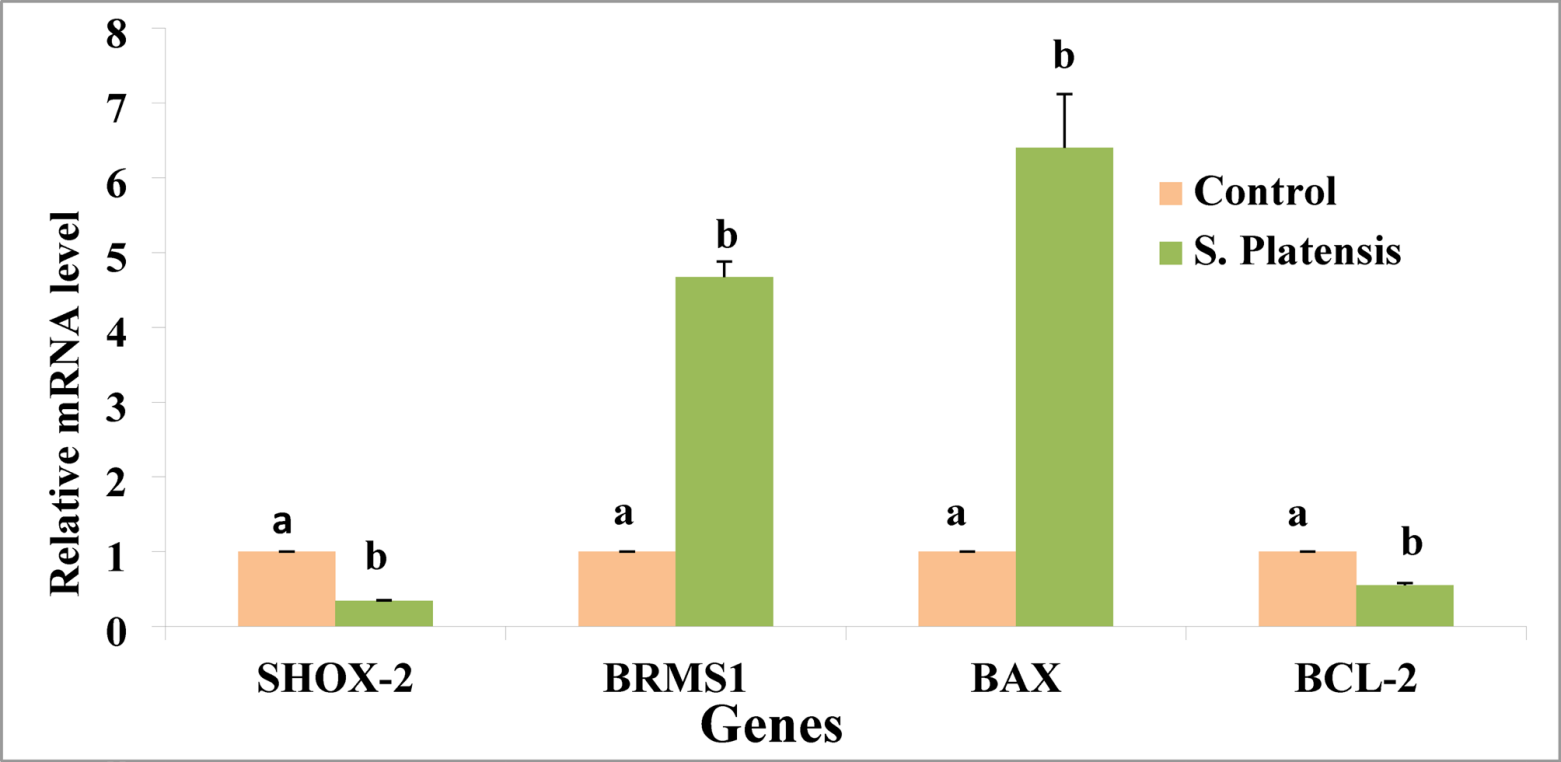
The relative mRNA expression level of SHOX-2, BRMS1, BAX and BCL-2 in the control A549 and the treated cell line with *S. platensis.*

The *S. platensis* provided potential anti-proliferative effects on the A549 lung cancer cell line, such that significant up-regulations of the BRMS1 and BAX were reported. On the other hand, downregulations of both the SHOX-2 and BCL-2 were recorded in our findings. Figure 6.

### Molecular docking

#### Docking simulation and docking

**Fig. 7:**
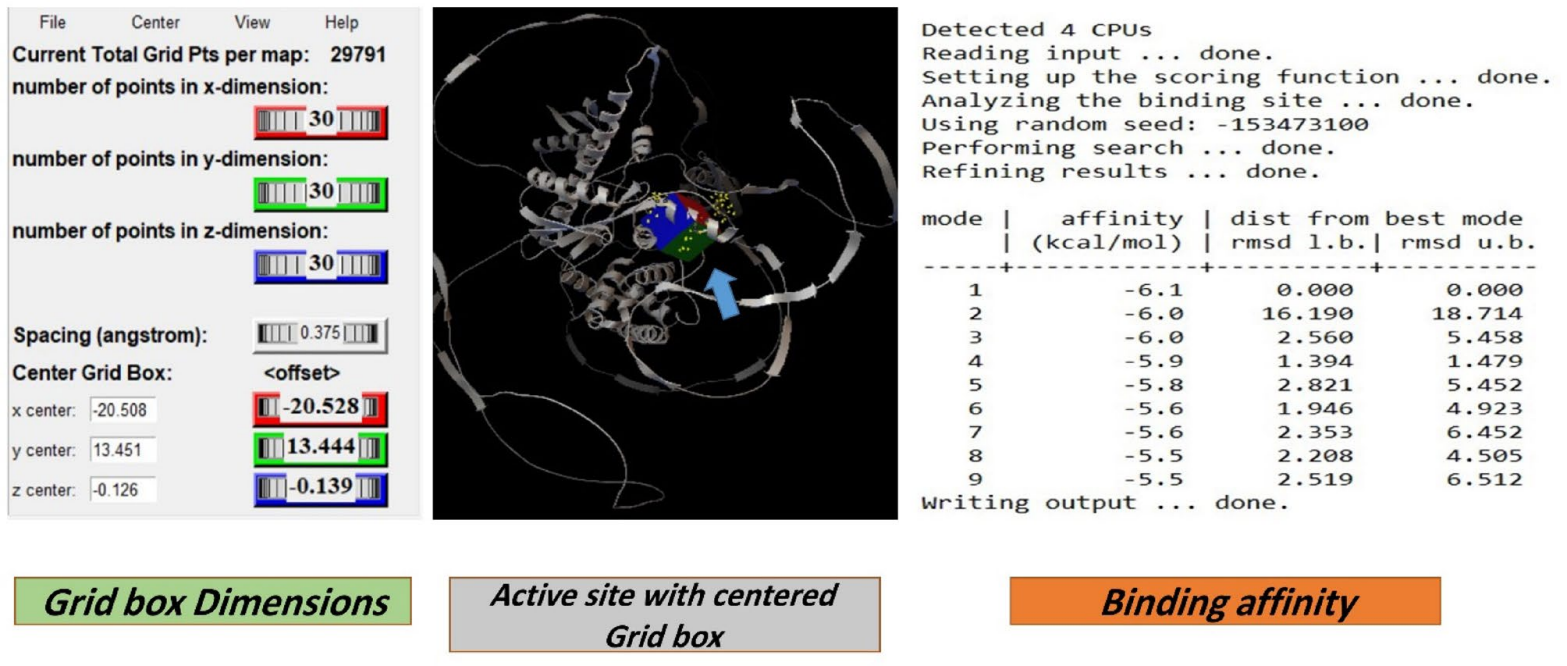
Active site and Grid box dimensions with the binding affinity between *S. platensis* & target receptor with energy binding of -6.1 Kcal.mol.

**Fig. 8:**
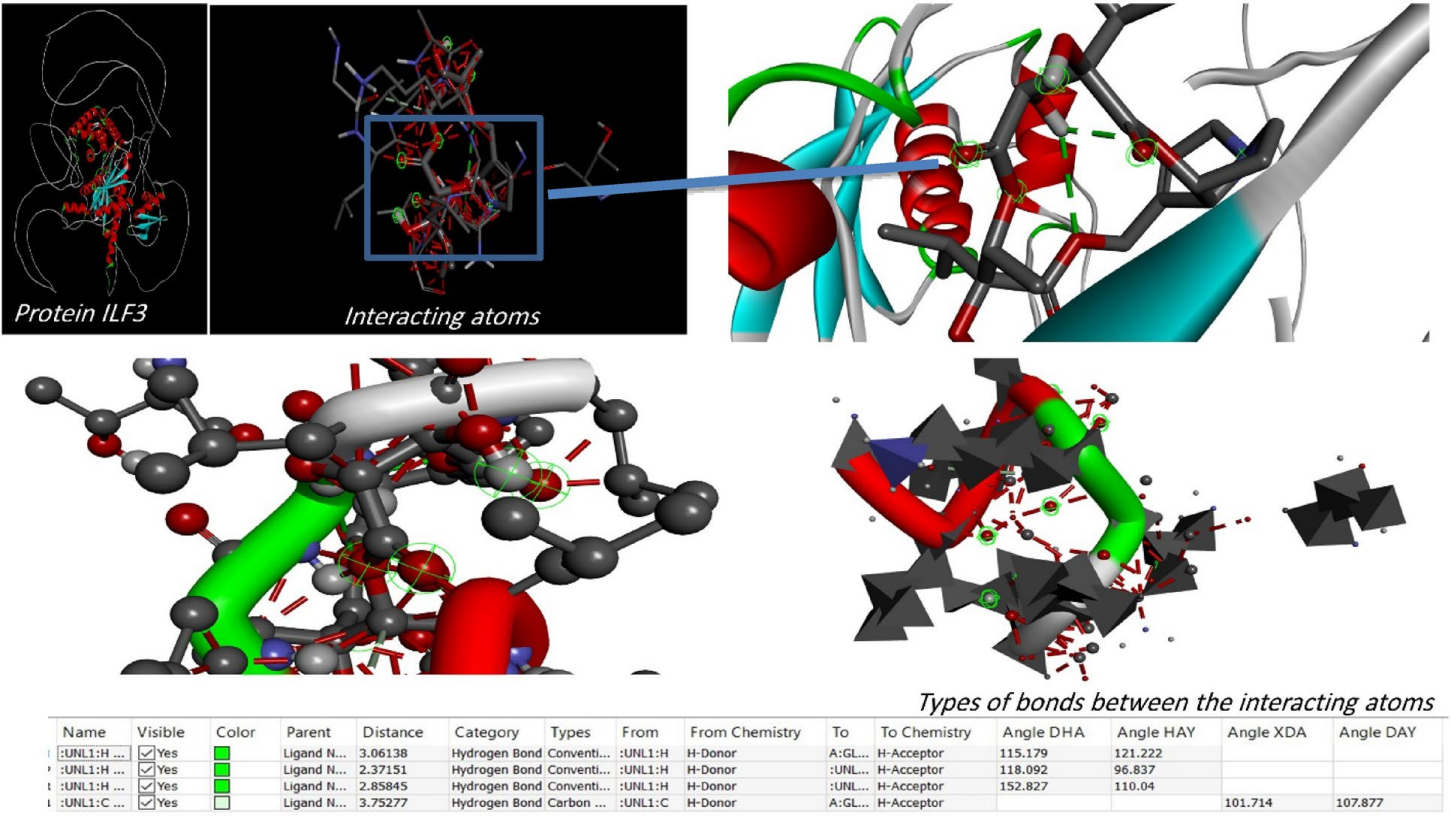
Binding site and key amino acid of interaction or binding between *s.platensis* & ILF3 receptor. Carbon, hydrogen, oxygen, nitrogen and sulfur atoms are in grey, white, red, and blue colors, respectively; H-bonds are presented in dotted lines are presented.

On the contrary, docking experiments revealed that the predicted active sites of *S. platensis* and the ILF2 receptor on the surface of lung carcinoma exhibited the maximum binding affinity, as illustrated in Fig. 7, in comparison to carboplatin at the first active site identified for ILF2. The binding affinities of the ligand and receptor at the determined active site were (-6.1 kcal/mol), which is significantly lower than the binding affinities of the standard carboplatin drug at the active site (-5.1 kcal/mol) (Fig. 11). Based on these findings, *S. platensis* and the ILF2 receptor on the surface of lung cancer interact *via* a variety of hydrogen bonding interactions. As pathway MAP will demonstrate, the functional enrichment analysis identified a number of interactions between overrepresented protein domains in the network, which may indicate the presence of particular biological functions or pathways that are active in the system. Hydrophobic and H-bonding interactions are present in the majority of complexes. H-bonding was observed between the active side residues of the amide oxygen or nitrogen of the majority of compounds, including 101Gly, 103Lue, 358Gln, 356Thr, 102Leu, 100Lys, 97Leu, 100Lys, 199Ala, and 101Gly. An attraction towards salt bridges is discernible at the site of interaction or between acidic amino acids and nitrogen (Figs. 8 and 10). The binding site and the proximity of the amino acids to their bonded ligand atoms, as well as the formed bonds that are illustrated in various positions, are also illustrated. Table 2 displays the smiles codes, as well as the ligand, protein structure, and bond types.

**Fig. 9:**
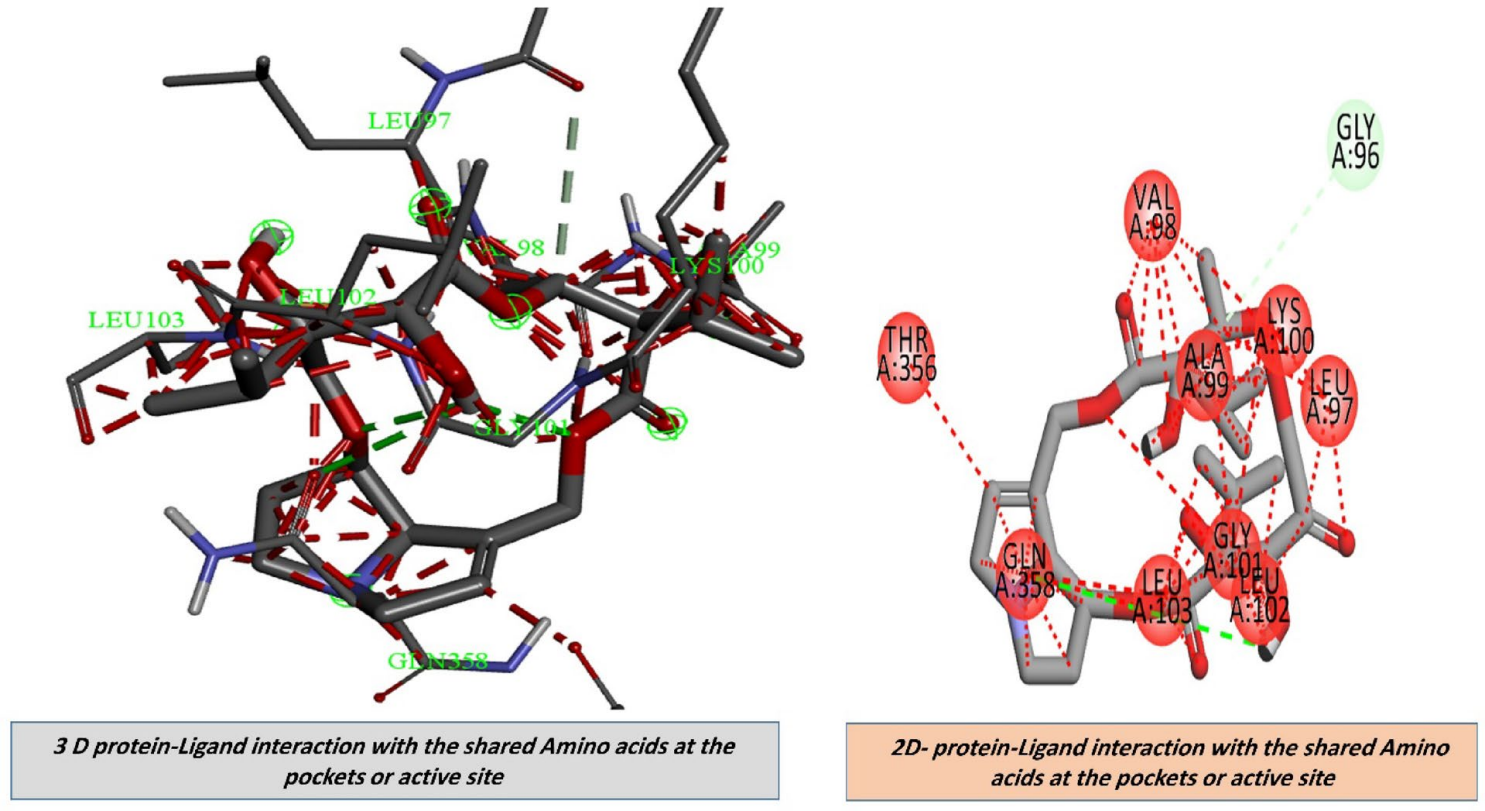
3D and 2D representations of the active sites, as seen using the BIOVIA discovery studio.

**Fig. 10:**
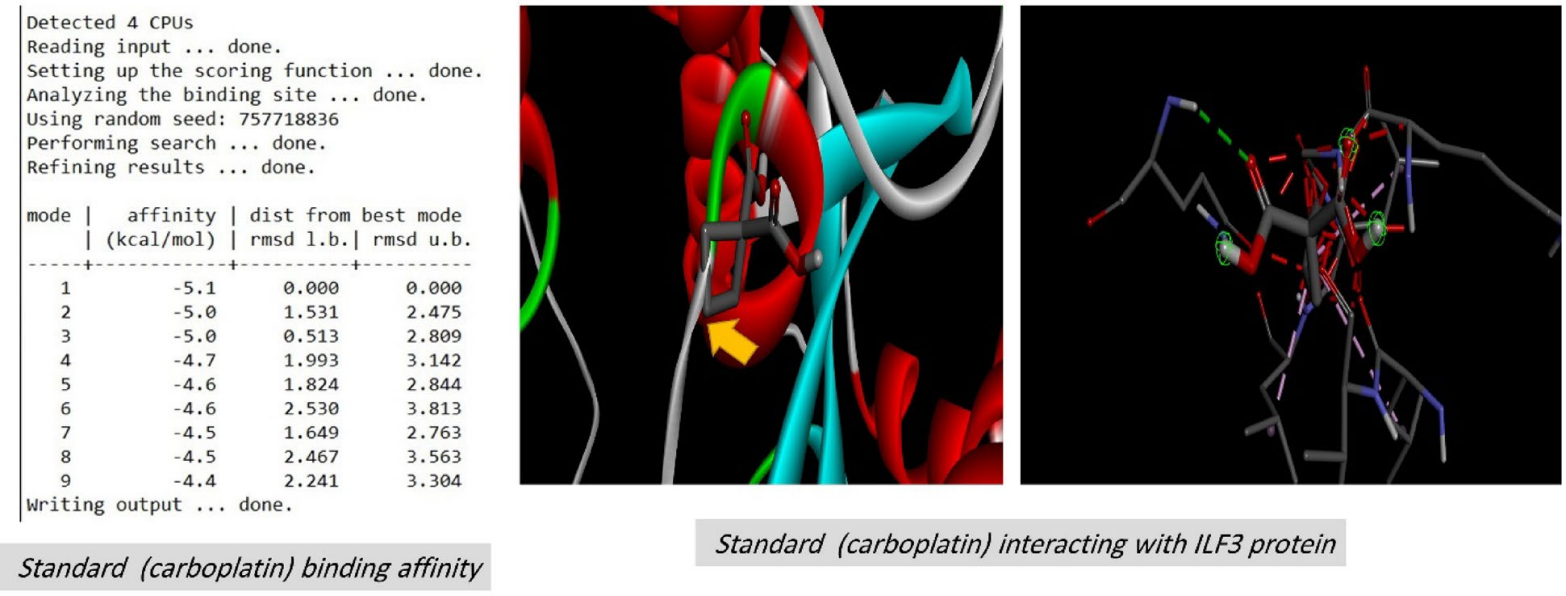
Binding affinity between carboplatin standard drug & target receptor with energy binding of -5.1 Kcal.mol.

**Fig. 11:**
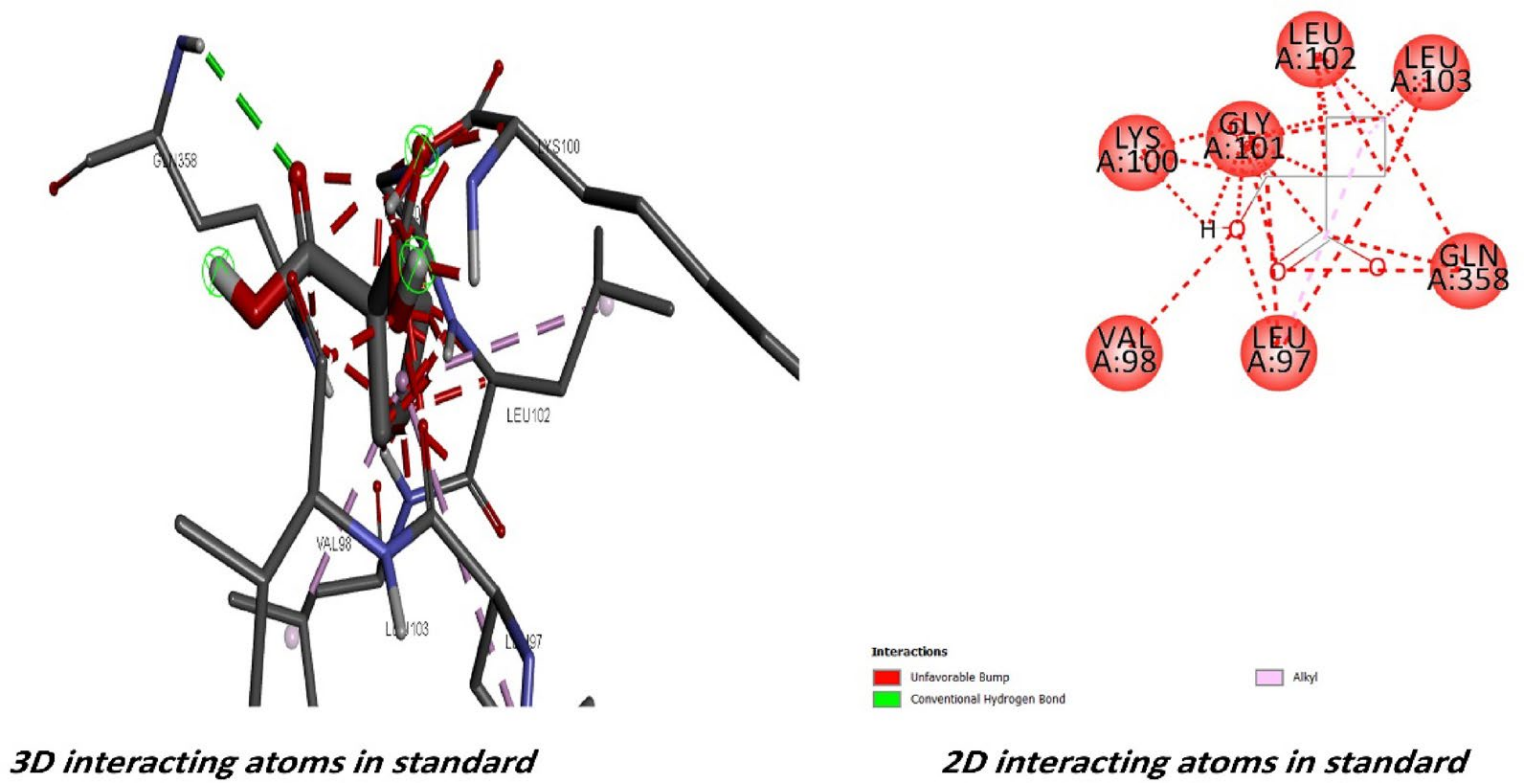
3D and 2D representations of the active sites, as seen using the BIOVIA Discovery Studio for the compared standard drug.

The interactions between the bound ligand and the receptor were illustrated in Figs. 7, 8, 9, 10 and 11 for the standard drug and S. platensis, respectively. The interaction between VAL and LUE amino acids and receptor residues is predominantly mediated through hydrogen bonding; however, ligands have the ability to establish pi–pi stacking interactions with the side chains of the C-terminal GLN and ELU residues.

**Table 2:** Binding affinity, the total number and sites of hydrogen bonds, and pi-pi stacking formed between the ligands and the protein residues at the ILF3 binding domain (other parameters are presented in Fig. 8):

No.	Ligand compound	Binding affinity (site 1) Kcal/ mol	PDB ID	Bond interactions	Bond Type	Smiles code
				Bond type		
1	Spiraline	-6.1	-	H-Bond	Conventional Hydrogen Bond	CC1C(C(= O)OCC2 = CCN3C2C(CC3)O C(= O)C(C(C(= O)O1)O)(C(C)C)O)(C(C)C)O
2	Carboplatin	-5.1	-	H-Bond	Conventional Hydrogen Bond	C1CC (C1) (C (= O)O)C(= O)O.[NH2-]. [NH2].[Pt + 2]
3	ILF3		Q12906 · ILF3_HUMAN			

#### Prediction of ADMET properties

**Fig. 12:**
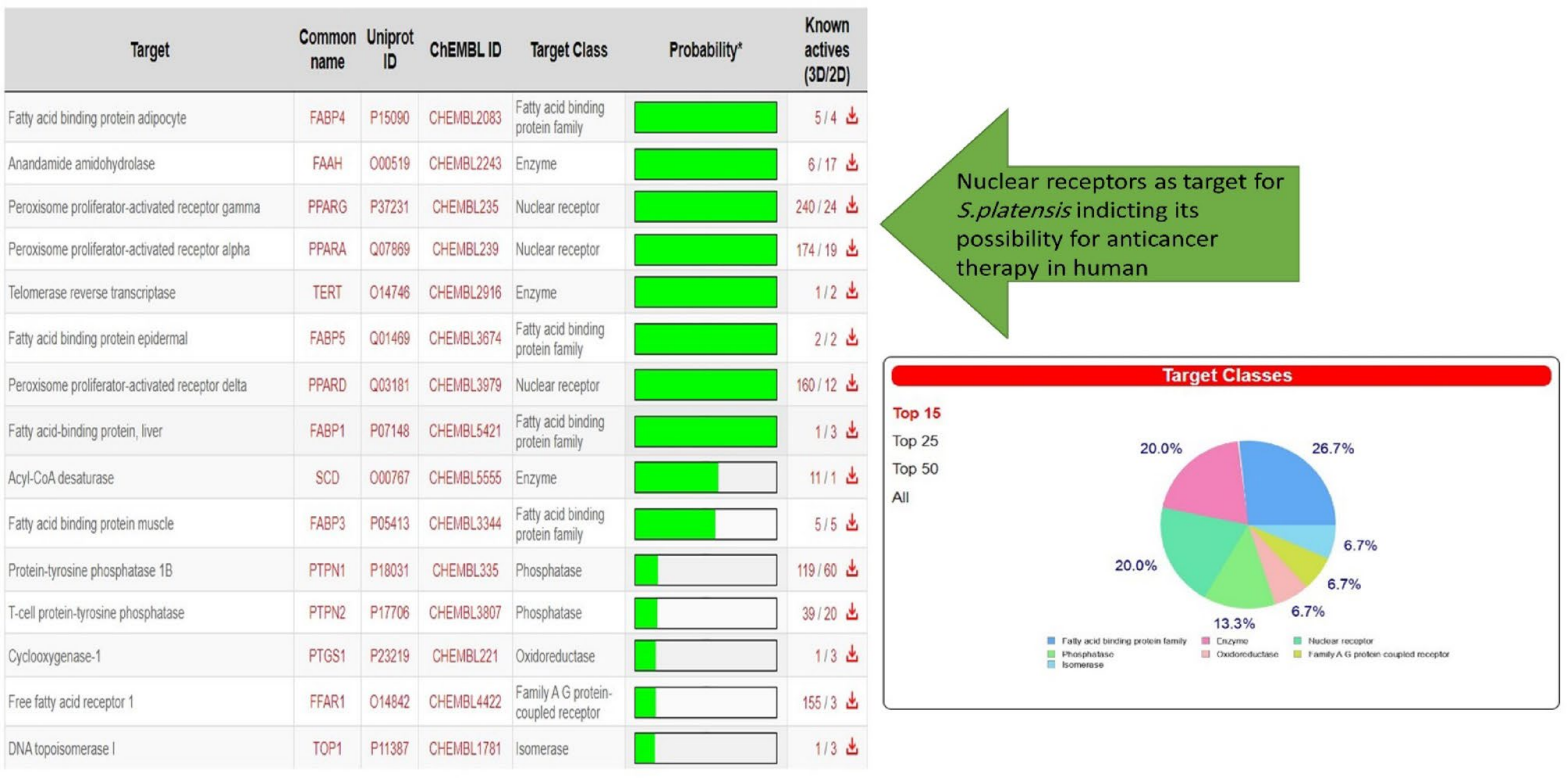
Predicted target site of *S. platensis* against human proteins with high percentages towards to the nuclear receptors.

As illustrated in Fig. 12, S.*platensis* was targeted following nuclear receptor prediction. In Table 3, the ADMET properties of S.platensis are detailed. *S. platensis* exhibited a PSA_3D value exceeding 140, indicating that these compounds possessed substantial polarity and were resistant to hepatic absorption. A value below 100 would suggest that *S. platensis* exhibited favorable oral absorption or membrane permeability^47^. Ideal lipophilicity was predicted for S.platensis (AlogP98 < 5); inadequate lipophilicity was indicated by (AlogP98 > 5) ^48^. Intestinal absorption (human), Caco-2 permeability, cutaneous permeability, and P-glycoprotein substrate or inhibitor were utilized to forecast the S.platensis absorption level. In the case of intestinal absorption (human), a compound is deemed to have high Caco-2 permeability and is easily absorbed when the predicted value of the Papp coefficient is greater than 0.90. Absorbance values below 30% indicate inadequate absorption. It was anticipated that *S. platensis* would have little absorption. In terms of dermal permeability, S.platensis is characterized by a relatively low log Kp value of 2.5 or greater. As a member of the ATP-binding transmembrane glycoprotein family (ATP-binding cassette (ABC)), P-glycoprotein is capable of eliminating exogenous compounds and pharmaceuticals from cells. The findings indicated that all of the S.platensis components are substrates of P-glycoprotein and that P-glycoprotein may actively expel them from the cells.

To characterize the distribution of compounds, the fraction unbound (human), CNS permeability, blood-brain barrier membrane permeability (logBB), and distribution volume (VDss) were utilized. As a parameter, distribution volume characterizes the in vivo distribution of medications across a variety of tissues. A VDss value below 0.71 (log VDss = 0.267 log L/kg) indicates a relatively small volume of distribution. As VDss exceeds 2.81 log L/kg, the volume of distribution is deemed to be relatively substantial. The findings indicated that the volume of S.platensis distribution was minimal. In the context of blood-brain barrier membrane permeability, *S. platensis* was hypothesized to readily traverse the barrier (logBB > -0.7). A logBBB value below 1 indicates that the compounds encountered difficulty traversing the blood-brain barrier. S.platensis was anticipated to traverse the blood–brain barrier with great difficulty.

In terms of CNS permeability, it was hypothesized that *S.platensis* would be incapable of infiltration (logPS < -3). Cytochrome P450s are an essential system of enzymes for the hepatic metabolism of drugs. CYP2D6 and CYP3A4 are the two primary subtypes of cytochrome P450 that are unaffected by *spirulina*. *S.platensis* was not identified as a substrate by either of the two subtypes; this indicated that the organism might not undergo hepatic metabolism. Compounds’ molecular weight and hydrophilicity influence drug elimination. The prediction results indicate that S.platensis will be completely cleared; the results also indicate that it will not be toxic in the AMES test; *S.platensis* may not be hepatotoxic; and it did not cause cutaneous sensitization or cardiotoxicity.

Therefore, the anticipated outcomes suggest that the ADMET properties of *S. platensis* are comparable to those of conventional carboplatin. Nevertheless, the clearance and metabolism of S.platensis were impeded due to its high lipophilicity. It can traverse the blood-brain barrier with relative ease and may be AMSE toxic. This warrants considerable attention. Moreover, due to its high lipophilicity, it is capable of traversing the blood-brain barrier and causing neurotoxicity. Each of these parameters is detailed in Table 3.

**Fig. 13:**
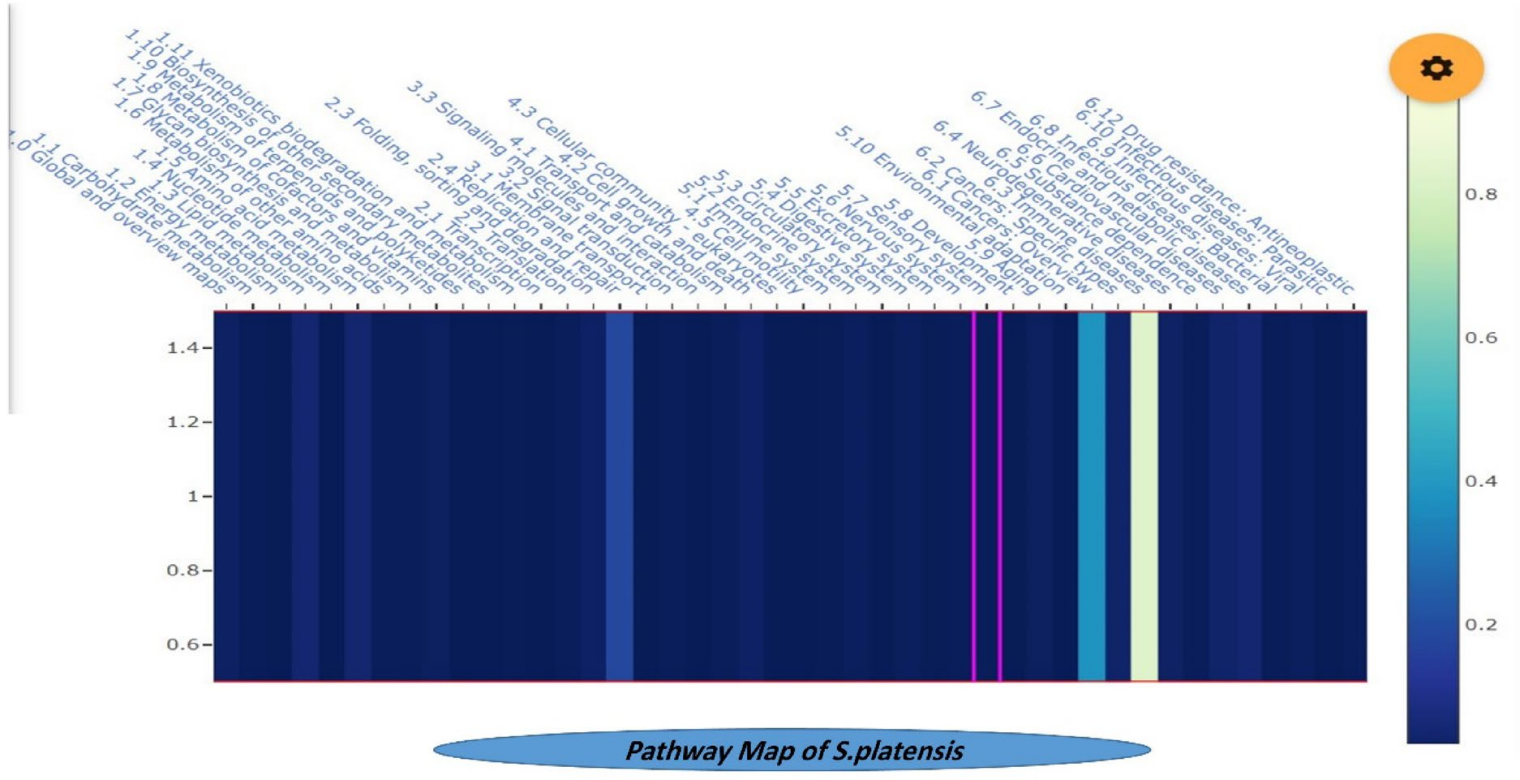
Pathway Map of *S. platensis* on different body systems according to the probability difference.

**Fig. 14:**
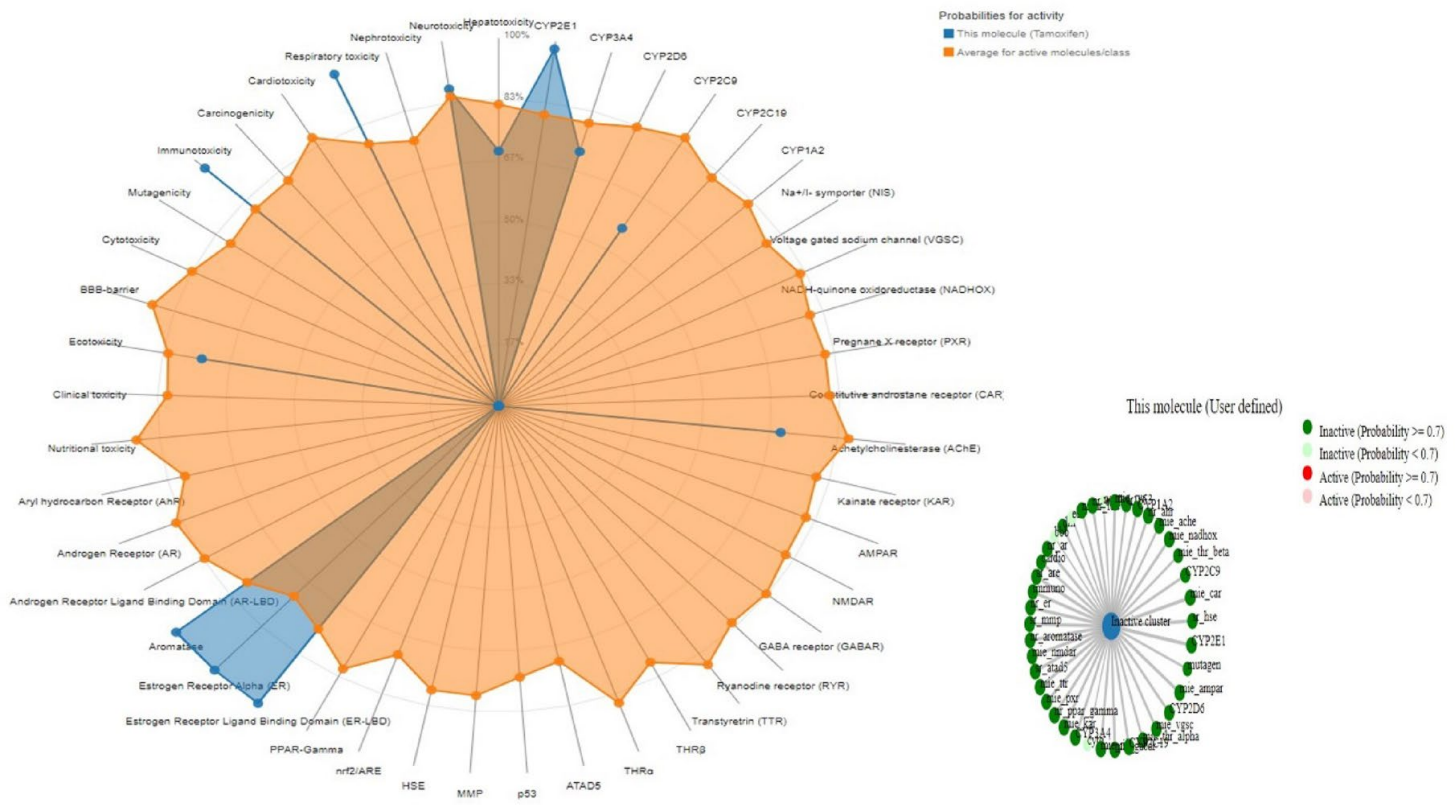
Activity of *S. platensis* on different body organs and enzymes (safety investigations).

**Fig. 15:**
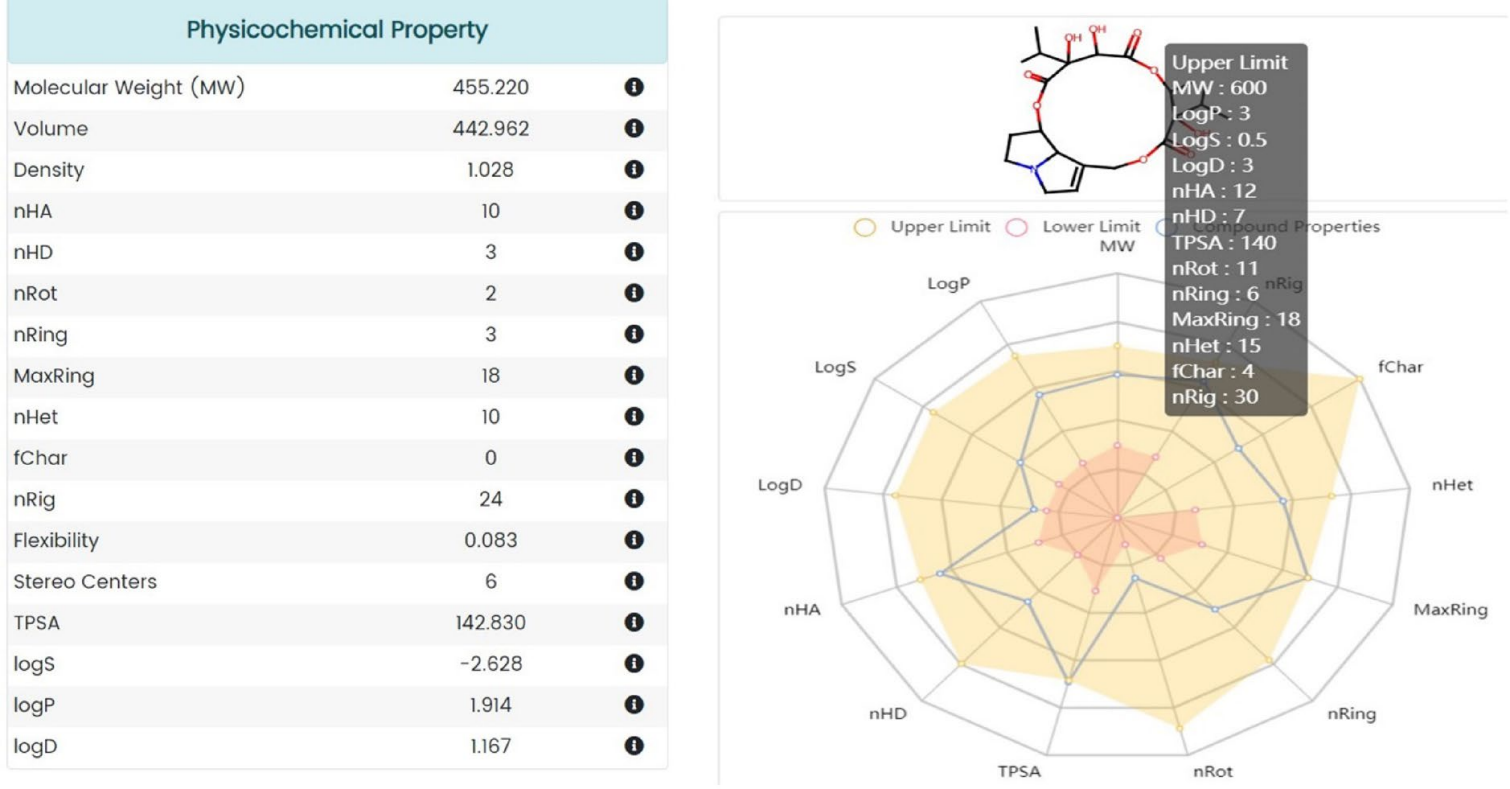
Physiochemical properties of *S. platensis.*

Figure 13 illustrates the pathway MAP of *S. platensis* on various body organs, Fig. 14 depicts safety parameters, and Fig. 15 illustrates physiochemical properties.

**Table 3:** Predicted ADMET properties of compounds:

Properties	S. platensis	Standard (Carboplatin)
PSA	186.664	
LogP98	1. 94	-2.19
Lipinski rule	Accepted for druglikness	Accepted for druglikness
Absorption		
Water solubility (log mol/L)	-3.286	-1.92
Caco2 permeability (log Papp in 10^− 6^ cm/s)	0.799	-6.92
Intestinal absorption (human) (% absorbed)	50.383	27.654
Skin permeability (log Kp)	-2.754	-2.735
P-Glycoprotein substrate	Yes	No
P-Glycoprotein I inhibitor	No	No
P-Glycoprotein II inhibitor	No	No
Distribution		
VDss (human, log L/kg)	0.267	-1.2
Fraction unbound (human) (Fu)	52.67% (0.463)	0.511
BBB permeability (logBB)	-0.714	-0.433
Metabolism		
CYP2D6 substrate	No	No
CYP3A4 substrate	No	No
CYP1A2 inhibitor	No	No
CYP2C19 inhibitor	No	No
CYP2C9 inhibitor	No	No
CYP2D6 inhibitor	No	No
CYP3A4 inhibitor	No	No
Excretion		
Total clearance (log ml/min/kg)	0.212	0.848
Renal OCT2 substrate	No	No
Toxicity		
AMES toxicity	No	No
hERG I inhibitor	No	No
Hepatotoxicity	No	No
Skin sensitization	No	No

ADMET, absorption, distribution, metabolism, excretion, and toxicity; Papp, apparent permeability coefficient; AMES, assay of the ability of a chemical compound to induce mutations in DNA; Kp, skin permeability constant; Fu, fraction unbound; BBB, blood–brain barrier; BB, blood–brain; CNS, central nervous system; PS, permeability-surface area; T. pyriformis, Tetrahymena pyriformis; LD, lethal dose; LOAEL, lowest-observed-adverse-effect level.

## Discussion

*S. platensis* is a natural algal extract with abundant content of micronutrients, proteins, and phytopigments as (carotenoids, chlorophyll, phycocyanin^49^. Phycocyanin may be attributed to the anti-carcinogenic effect of *Spirulina*, it could disrupt the genomic DNA synthesis with in the cancerous cell^50^, or stimulating the cellular apoptotic signal transduction through stimulating the surface expression of CD59 following binding to the mitogen receptor on cancerous cell^51^, Or promotion of the cellular death through up-regulation of both Bax and BAD resulting in mitochondrial dysfunction^52^. *Spirulina* contains another bioactive compound called b- b-carotene^53^, which proves to have prominent protective and preventive effect against skin^54^ and cervical cancers^32^. B-carotene stimulates the body to produce signals the cancerous line to end the dividing process through opening the membrane communication channels of the cancerous and pre-cancerous cells^55^. These effects were investigated on many cancerous cell lines, confirming their anti-proliferative, anti-cancerogenic, and pro-apoptotic properties^50^.

*S. platensis* has been shown to possess various health benefits, including anti-cancer properties. Recent studies have demonstrated the anti-proliferative cytotoxic effect of *S. platensis* against A549 lung cancer cell line^56^.

In the current study, *S. platensis* exerts a great anti-proliferative cytotoxic effects on A549 cell line that come in accordance with^2^, and^56^. Furthermore, *Spirulina* extracts exerted cytotoxic activity against CM human chronic myelogenous^22^, K-562 chronic myelogenous leukemia and Kasumi-1 human acute leukemia^21^, HEPG2 hepatocellular and HCT116 colon carcinoma^20^. ^57^ was noted that the origin of tumor cells greatly affects on the *spirulina*^,^ cytotoxic effects through changing the cellular permeability of the tumor cells, facilitating the infiltration of the bioactive compounds during cultivation with the tested *spirulina* extract. The antioxidant property of *Spirulina* is also related to its phytopigments content^49^, tocopherols, and phenolic acids, through marked elevation the antioxidant-related enzymes activity such as catalase, glutathione peroxidase and reductase, and superoxide dismutase^58^. Our study revealed that *S. platensis* causes a significant elevation of the total thiol contents and lowers the MDA content that come in accordance with^59^ and in contrast with^56^.

A549 lung cancer cell line is a widely used pre-clinical model to evaluate the anti-cancer effects of various compounds^60^. This effect has been attributed to the modulation of various genes involved in the regulation of cell proliferation and apoptosis.

To investigate the molecular mechanisms underlying the anti-cancer effects of *S. platensis*, several genes were evaluated, including SHOX2, BRMS1, BAX, and BCL2. The current results showed that *S. platensis* down-regulated the expression of SHOX2 and up-regulated the expression of BRMS1 in A549 lung cancer cells. One of the genes that has been shown to be modulated by *S. platensis* is SHOX2, which is a tumor suppressor gene that inhibits cell proliferation and induces apoptosis. It is involved in the regulation of cell proliferation and differentiation, and its dysregulation has been implicated in the development of various cancers^61^. *S. platensis* has been shown to down-regulate the expression of SHOX2 in A549 lung cancer cells, which may contribute to its anti-proliferative effect.

BRMS1 is a metastasis suppressor gene that inhibits the spread of cancer cells to other parts of the body. It is involved in the regulation of metastasis, and its down-regulation has been associated with increased metastatic potential in various cancers ^62,63^. *S. platensis* has been shown to up-regulate the expression of BRMS1 in A549 lung cancer cells, which may contribute to its anti-metastatic effect.

The BAX and BCL2 genes are also involved in the regulation of apoptosis, which is a key process in the development and progression of cancer. BAX promotes apoptosis, while BCL2 inhibits apoptosis^64^. *S. platensis* has been shown to up-regulate the expression of BAX and down-regulate the expression of BCL2 in A549 lung cancer cells, which may contribute to its pro-apoptotic effect^65^. Furthermore, *S. platensis* induced cell cycle arrest at the G0/G1 phase, which is a hallmark of anti-proliferative effects^66^.

In carcinogenesis, early epigenetic changes have been reported and become evident in the precursor lesions of lung^67^, breast^68^, endometrium^69^, and colon^70^ cancers. Gene methylation has been recorded in patients suffering from lung cancer with a smoking history. In lung adenocarcinomas/squamous cell carcinomas, the frequency of p16 promoter methylation was markedly greater in smokers than non-smoker patients^71^. Moreover, P16 is frequently mutated or deleted in NSCLC^72^. Cigarette smoking results in chronic inflammation, which has a major role in the progression of lung cancer, proliferation, and stimulating the cellular turnover. In lung cancer, there is a great link between DNA methylation and inflammation^73^. On the other hand, the generated reactive oxygen species (ROS) during the chronic inflammation and the target transcriptional repressors are accelerating the levels of DNA methylation^74^.

In the current study, the concentration of P16 was significantly decreased, which may be due to the decreased lipid peroxidation content following *S. platensis* therapy.

According to our research, A549 cells treated with *S. platensis* had much lower concentrations of MTUS1 and P16 proteins than A549 cells that were not treated. This shows that the expression or stability of these proteins in lung cancer cells may be affected by the *S. platensis* treatment.

MTUS1 (also known as ATIP) is a multifunctional protein that has been implicated in various cellular processes, including cell proliferation, migration, and invasion^75^. It acts as a tumor suppressor by inhibiting key signaling pathways involved in cancer progression^76^. Decreased levels of MTUS1 have been associated with poor prognosis and increased tumor aggressiveness in several cancer types. In the context of *S. platensis* treatment, the observed low concentration of MTUS1 in A549 cells may suggest a potential mechanism by which *S. platensis* exerts its anti-proliferative effects.

P16 (also known as CDKN2A or INK4A) is another well-known tumor suppressor protein involved in cell cycle regulation^77^. It inhibits the activity of cyclin-dependent kinases (CDKs), preventing cell cycle progression and cell proliferation. Loss or decreased expression of P16 is frequently observed in various cancers, including lung cancer, and is associated with uncontrolled cell growth^78^. The significant decrease in P16 protein concentration following *S. platensis* treatment in A549 cells suggests that *S. platensis* may modulate the cell cycle machinery and inhibit cell proliferation.

Westcott and his colleagues recorded the mutational landscapes of KRAS driven lung cancer, knockdown of MTUS1 expedited growth in a mouse lung cancer cell line caused by KRAS G12D^79^. Inhibition of DNA methylation in the A549 cell line with 5-aza-20- deoxycytidine causes significant up-regulation of MTUS1 expression, therefore MTUS1 may be encoded by DNA methylation^80^. This mechanism was markedly noted in the present study in which P16 and K-ras was significantly down-regulated because of *S. platensis* and subsequently MTUS1 was down-regulated.

Moreover, the protein concentration of K-ras and EGFr was observed to be significantly reduced in the *S. platensis*-treated A549 cell line. K-ras and EGFr are known to play important roles in cell signaling and proliferation ^81,82^, and their reduction suggests that the treatment with *S. platensis* may have an inhibitory effect on these pathways in the A549 cells.

K-ras (Kirsten rat sarcoma viral oncogene homolog) is a member of the Ras protein family and is a key component of the Ras/ERK signaling pathway^83^. This pathway is crucial for regulating cell growth, survival, and differentiation. Mutations in the K-ras gene are commonly found in various cancers, including lung cancer, and can lead to constitutive activation of the pathway^84^. The observed reduction in K-ras protein concentration in *S. platensis*-treated A549 cells suggests that *S. platensis* may interfere with the Ras/ERK signaling pathway, potentially inhibiting cell proliferation and promoting tumor suppression.

EGFr (epidermal growth factor receptor) is a cell surface receptor that plays a vital role in cell signaling and is frequently overexpressed in cancer cells^82^. EGFr activation triggers downstream signaling cascades that promote cell growth, survival, and metastasis^85^. Inhibition of EGFr signaling has been a successful therapeutic strategy in several cancers^86^. The significant reduction in EGFr protein concentration following *S. platensis* treatment suggests that *S. platensis* may interfere with EGFr signaling, leading to decreased cell proliferation and potentially inhibiting tumor growth and progression.

These observed changes in protein levels provide valuable insights into the molecular mechanisms underlying the anti-proliferative effects of *S. platensis* in A549 lung cancer cells. By targeting key proteins involved in cell cycle regulation and cell signaling pathways, *S. platensis* may disrupt the growth and survival of cancer cells. Understanding the specific mechanisms by which *S. platensis* modulates these protein levels can potentially contribute to the development of novel therapeutic approaches for lung cancer treatment. The down-regulation of proteins such as K-ras and EGFr suggests that *S. platensis* treatment may inhibit the proliferation of A549 cells. The reduced expression of MTUS1 and P16, which are tumor suppressor proteins, may also contribute to the inhibition of cell growth.

Based on the results of the investigation, *S. platensis* exhibited a significant affinity for the active sites of ILF3 of the lung cancer protein receptor that were identified. The active site bound affinities were − 6.1% by mol, whereas the standard site bound affinities were − 5.1% by mol. These results suggest that *S. platensis* might be amenable to inhibition of lung cancer growth, particularly following the identification of the nuclear receptors it targets. It was determined, upon further examination of the interactions with the active sites, that the ligand of active site 1 formed alkyl bonds with the receptor over an average distance of angstroms. Alkyl interactions are typically feeble and occur between homologous, nonreactive carbon groups in organic molecules. Aromatic and aliphatic groups engage in pi-alkyl interactions, which are distinguished by the pi-electron density of the aromatic ring crossing with the electron density of the alkyl group. The weak, non-covalent van der Waals interactions, in addition to H. H-bonds, may account for a portion of the compound’s binding affinity, as suggested by these interactions. Hydrogen bond interactions are feeble interactions caused by variations in the electron density of atoms or molecules. The aforementioned interactions imply that alpha guanine might establish a stable complex with H. van der Waals forces and bonds.

The ADMET, in addition to the physical and chemical properties, is determined by the structure of a compound. Here, specific predictive software was used to analyze the ADMET parameters of S.platensis, and structural cautions for their potential genotoxicity were predicted using three toxicology databases. The docking outcomes indicate that the identified compounds may impede the tested target protein from culex via a variety of interaction mechanisms. As previously stated, S.platensis interacted with particular amino acids in the protein via hydrogen bonding and alkyl interactions, whereas the compound itself recognized interactions with numerous amino acids. The ΔG values, which represent the energy favorable nature of these interactions, are calculated. Additionally, the pKd values, which represent the differing degrees of binding affinity of the compounds towards the target proteins, are suggested.

All safety investigations on different body organs and enzymatic functions besides the pathway MAP for *S.platensis* with different probability if action on various body systems.

## Conclusion

In conclusion, our study reveals that *S. platensis* exerts an anti-proliferative cytotoxic effect on A549 lung cancer cells by influencing genes associated with cell proliferation, metastasis, apoptosis, and by increasing antioxidant levels. However, further investigation is required to fully understand the molecular mechanisms that underlie the anti-cancer properties of *S. platensis* and to assess its potential as a therapeutic option for lung cancer. The inhibition affinity (pKd and ΔG) of *S. platensis*, which was recently crystallized via X-ray diffraction, was assessed in relation to the lung cancer receptor ILF3. *S. platensis* exhibited a significantly higher affinity for the receptor than carboplatin, the standard medication utilized in the treatment of lung cancer. The analysis revealed that the active site contained the following amino acid residues that were crucial for binding: (P: 174; R: 177; M: 180; E: 181; Y: 784; L: 207 & R: 211; all in the A-chain). Hydrophobic interactions and H-bonding are involved in interactions. In addition, safety and toxicological investigations are anticipated at the site of interaction.

Depending on the current results, future inspections and studies are recommended to study the anti-cancerous effects of *S. platensis* in relation to other natural or synthetic formulas used in cancer therapy and identifying the future limitations.

## Supplementary Information

Below is the link to the electronic supplementary material.

Supplementary Material 1

Supplementary Material 2

Supplementary Material 3

Supplementary Material 4

## Data Availability

All data generated or analysed during this study are included in this published article.

## Abbreviations

A 549: Adenocarcinomic human alveolar basal epithelial cells
ABC: ATP-binding cassette
ADMET: Absorption, distribution, metabolism, excretion, and toxicity
BAX: Bcl-2-associated X protein
BCL-2: B-cell lymphoma 2
BRMS1: Breast cancer metastasis suppressor 1
EGFr: Epidermal growth factor receptor
K-ras: Ki-ras2 Kirsten rat sarcoma viral oncogene homolog
MTUS1: Microtubule-associated tumor suppressor 1
PDAC: Pancreatic ductal carcinoma
PSA: Polar surface area
*S. platensis*: *Spirulina platensis*
SHOX2: Short stature homeobox 2
VDss: Distribution volume
