# Supplementary figures and images for: In vitro and in silico analysis of the anti-proliferative effects of Spirulina platensis on A549 lung cancer cells
Source: Sci Rep. 2025 Nov 7;15:39006. doi: 10.1038/s41598-025-24051-2 (PMC12594977; doi:10.1038/s41598-025-24051-2)

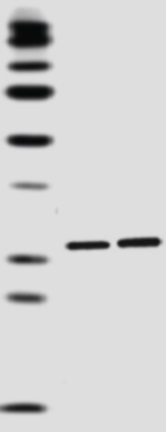

Supplement: Supplementary file 1 — Supplementary Material 1 [file 41598_2025_24051_MOESM1_ESM.jpg]

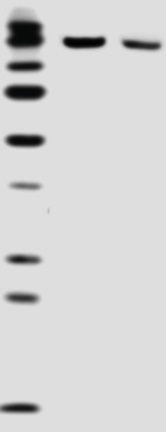

Supplement: Supplementary file 2 — Supplementary Material 2 [file 41598_2025_24051_MOESM2_ESM.jpg]

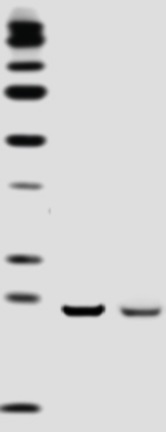

Supplement: Supplementary file 3 — Supplementary Material 3 [file 41598_2025_24051_MOESM3_ESM.jpg]
