# Supplementary material for: In vitro and in silico analysis of the anti-proliferative effects of Spirulina platensis on A549 lung cancer cells
Source: Sci Rep. 2025 Nov 7;15:39006. doi: 10.1038/s41598-025-24051-2 (PMC12594977; doi:10.1038/s41598-025-24051-2)
Supplement: Supplementary file 4 — Supplementary Material 4 [file 41598_2025_24051_MOESM4_ESM.docx]

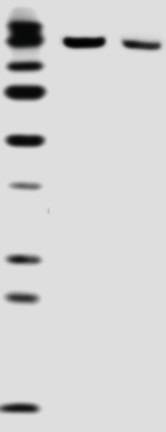


Fig. (S1): blot/gel image of EGFr concentration in the control A549 and the treated cell line with *S. platensis*.


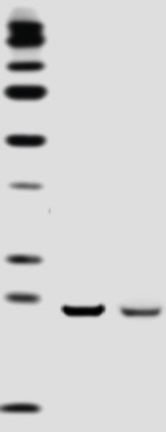


Fig. (S2): blot/gel image of k-ras concentration in the control A549 and the treated cell line with *S. platensis*.
